# Effect of methanolic extract from *Capsicum annuum* against the multiplication of several *Babesia* species and *Theileria equi* on *in vitro* cultures, and *Babesia microti* in mice

**DOI:** 10.14202/vetworld.2022.76-82

**Published:** 2022-01-20

**Authors:** Mohamed Abdo Rizk, Shimaa Abd El-Salam El-Sayed, Mostafa Al-Araby, Ikuo Igarashi

**Affiliations:** 1 National Research Center for Protozoan Diseases, Obihiro University of Agriculture and Veterinary Medicine, Inada-Cho, Obihiro, Hokkaido 080-8555, Japan; 2 Department of Internal Medicine and Infectious Diseases, Faculty of Veterinary Medicine, Mansoura University, Mansoura 35516, Egypt; 3 Department of Biochemistry and Chemistry of Nutrition, Faculty of Veterinary Medicine, Mansoura University, Mansoura 35516, Egypt; 4 Department of Parasitology, Faculty of Veterinary Medicine, Mansoura University, Mansoura 35516, Egypt.

**Keywords:** *Babesia*, *Capsicum annuum*, combination therapy, *in vitro*, *in vivo*, *Theileria*

## Abstract

**Background and Aim:**

Piroplasmosis is a serious disease that infects animals, inflicting significant economic losses in the livestock industry and animal trade worldwide. Anti-piroplasm drugs now on the market have demonstrated host toxicity and parasite resistance. As a result, developing more effective and safer anti-piroplasm drugs becomes an urgent issue. This study aimed to evaluate the inhibitory effect of *Capsicum annuum* methanolic extract (CA) against the growth of *Babesia bovis*, *Babesia divergens*, *Babesia caballi*, and *Theileria equi* *in vitro* and against *B. microti* in mice.

**Materials and Methods:**

Fluorescence-based SYBR Green I assay was used to evaluate CA’s inhibitory effect *in vitro* and *in vivo* when used either as a monotherapy or combined with diminazene aceturate (DA). The hematological parameters (HCT, hemoglobin, and red blood cells counts) were determined in the blood of mice every 96 h using Celltac a MEK-6450 electronic hematology analyzer.

**Results:**

The *in vitro* growth of *B. bovis*, *B. divergens*, *T. equi*, and *B. caballi* was inhibited by CA in a dose-dependent manner with IC_50_ values of 4.87±1.23, 44.11±8.03, 8.23±2.54, and 1.26±0.50 mg/mL, respectively. In *B. microti*-infected mice, a combination therapy consisting of CA and a low dose of DA showed a significant (p<0.05) inhibition of *B. microti* growth nearly similar to those obtained by treatment with the full dose of DA.

**Conclusion:**

The obtained results indicate that CA might be a promising medicinal plant for treating babesiosis, especially when used with a low dose of DA.

## Introduction

Tick-borne parasites *Babesia* and *Theileria* infect the erythrocytes of animals, causing major economic losses in the livestock industry and animal trade worldwide [1]. Fever, malaise, jaundice, hemoglobinuria, and mortality are among the condition’s symptoms [2]. The most common sources of infection in cattle are *Babesia bovis* and *Babesia divergens*, which result in severe losses in animal health and productivity [2]. *Theileria equi* and *Babesia caballi* are the most common causes of equine illness [3]. There are no acceptable laboratory experimental animals for bovine Babesia and equine piroplasm infections. Alternatively, a rodent *Babesia* model with *B. microti* is used to evaluate antibabesial drugs against babesiosis. The inhibitory effect of recently developed drugs should be evaluated in laboratory animals to identify the possible side effects before they are implemented in the field [4].

Anti-piroplasm medications currently on the market have shown toxicity to the host in the form of imidocarb dipropionate and resistance from the treated parasite in the form of diminazene aceturate (DA) [1]. Therefore, finding more effective and safer anti-piroplasm medicines becomes a priority. In this case, natural phytochemicals could be a potential alternative. Following this pattern, *Capsicum annuum* (CA), a member of the Solanaceae family, is generally considered safe for use in food by the US Food and Drug Administration [5]. CA has various medical and culinary applications [6]. Furthermore, it has been used as a food additive, as well as an antiseptic, counterirritant, appetite stimulator [6], antioxidant [7], and immunomodulatory [8] in traditional medicine for treating cough, toothache, sore throat, parasitic infections, rheumatism, and wound healing [6,9]. It has also been suggested to have antibacterial and anticancer properties [8]. In an *in vitro* research, extracts from the leaves of CA caused *Schistosoma mansoni* cercaria to die within 15 min. Water-soluble unsaturated molecules from oils or their hydrolysis products appeared to be the active factors [10].

There has been no previous research on the effectiveness of CA extracts as an anti-piroplasm agent. This study aimed to look into th*e* CA’s potential as an anti-piroplasm agent against the *in vitr*o development of bovine and equine *Babesi*a*/Theileri*a species, as well as *B. microt*i in mice.

## Materials and Methods

### Ethical approval

The stud was approved by the Animal Care and Use Committee at the Obihiro University of Agriculture and Veterinary Medicine (Approval No. 27-65). All experiments were carried out following the Ministry of Education’s Culture, Sports, Science, and Technology, Japan Fundamental Guidelines for the Proper Conduct of Animal Experiments and Related Activities at Academic Research Institutions.

### Study period and location

The study was conducted from July 2018 to November 2019 at National Research Center for Protozoan Diseases, Obihiro University of Agriculture and Veterinary Medicine, Japan.

### Chemicals

SYBR Green I (SGI) is a nucleic acid stain (Lonza, Rockland, USA; 10,000x). A lysis buffer was made ahead of time and kept at 4°C. CA powder was purchased from iherb.com. Methanol was 99.8% pure (Wako Pure Chemical Industries, Ltd., Osaka, Japan) by dissolving 100 mg (crude extract) in 1 mL of dimethyl sulfoxide (DMSO) (Wako Pure Chemical Industries, Ltd.), we were able to make a stock methanolic extract of CA solution. DA was bought from Ganaseg, Ciba-Geigy Japan Ltd., Tokyo, Japan.

### CA methanolic extract preparation

CA powder (10 g) was dissolved in 50 mL methanol and then incubated for 3 days at 30°C. After that, the resultant product was filtered using Whatman filter paper No. 1. The resulting extract was concentrated and lyophilized using a rotary evaporator (BUCHI® Rotavapor R-200/205, Flawil, Switzerland) under decreased pressure at 40°C and a freeze-dry vacuum system (Labconco, Kansas, MO, USA) [11,12].

### Determination of the toxic effect of CA methanolic extract on host erythrocytes

CA toxicity to host erythrocytes was assessed as previously described [13]. For 3 h at 37°C, bovine and equine erythrocytes were prepared with either media alone or media with CA methanolic extract (100 mg/mL). Then, using drug-free media, a triple wash cycle of red blood cells (RBCs) was conducted, followed by the cultivation of *Babesia* parasites in the washed RBCs for 72 h, and the parasite’s growth was evaluated in both pre-treated and control untreated cells. Each drug concentration was tested in three wells and three different trials for each parasite species.

### *In vitro* growth inhibition and viability assays

The chemotherapeutic efficacy of CA against *B. bovis* (Texas strain) [14], *B. divergens* (German strain) [15], *B. caballi* [3], and *T. equi* (US Department of Agriculture) [3] was studied. According to AbouLaila *et al*. [13] and Rizk *et al*. [14], parasites were cultivated in species-specific RBCs. CA’s capacity to inhibit *Babesia/Theileria* growth was investigated using a fluorescence assay and SGI stain [14,16]. Parasitized RBCs (pRBCs) were cultured with various *Babesia* species using media alone or with CA concentrations ranging from 0.5 to 100 mg/mL in two 96-well plates (Nunc, Roskilde, Denmark). M199 medium (Sigma-Aldrich, Tokyo, Japan) was used to culture *B. bovis* and *T. equi* parasites, while RPMI 1640 medium (Sigma-Aldrich) was used for *B. divergens* and *B. caballi*. A preliminary investigation was conducted to determine the concentrations. The commonly used antibabesial drug, DA was used as a positive control drug with concentrations ranging from 0.25 to 10 g/mL. Cultures without the medication and cultures containing only DMSO (0.3% for CA) and DDW (0.02% for DA) were generated as negative experimental controls. For each drug concentration, 96-well plates were used to cultivate RBCs parasitized with bovine and equine *Babesia/Theileria* parasites at 1% parasitemia using 2.5% HCT for *B. bovis* and 5% HCT for other *Babesia* and *Theileria* parasites for 4 days. On the 4^th^ day of culture, a 100 mL lysis buffer containing 2× SG I was added to each well on the first plate. Next, plates were covered with aluminum foil and incubated for 6 h at room temperature (34^o^C). A fluorescence plate reader (Fluoroskan Ascent; Thermo Labsystems, USA) was used for estimating the fluorescence values at 485 nm and 518 nm excitation and emission wavelengths. Gain values were set to 100. Finally, IC_50_ values were calculated. While the second plate was used to determine the regrowth of the tested parasites after discontinuing the *in vitr*o treatment using viability assay [15].

### CA and antibabesial drugs combination *in vitro*

Combining CA treatments with ordinarily utilized antibabesial drug, DA was evaluated against the *in vitro* cultures of *B. divergens* and *T. equi* (*Babesia* and *Theileria* spp. displayed the most elevated IC_50_ among screened parasites). As recently described, combined proportions for CA/DA (M1–M8) were set up [13,17]. Combination ratios were determined based on the IC_50s_. Drug-free cultures were employed as a negative control. Three independent trials were conducted, each comprising three triplicate studies with a 4-day medication combination utilizing 5% HCT. The fluorescence levels were calculated on the 4^th^ day of culture after adding lysis buffer to each medication combination on the 96-well plates as mentioned above.

### *In vivo* inhibitory effect of CA on the growth of *B. microti* in mice

The CA *in vivo* inhibition test for *B. microti* (Munich strain) [17] in BALB/c mice 2 months old (from CLEA Japan, Tokyo, Japan) was performed twice using a fluorescent-based SGI assay [18]. All mice were held under pathogen-free conditions. Twenty-five female BALB/c mice were divided equally into five groups. All mice were intraperitoneally infected with 1 × 10^7^ *B. microti* RBCs. Mice in the first group remained without infection and were used as the negative control. The parasitemia was observed in the infected mice, and once it reached around 1%, *B. microti*-infected mice received the treatment for 5 days. In the positive treatment control mice (second group), mice were infused with I/P dosages of DMSO in phosphate buffer saline (0.02%). DA was used as a control drug and administrated subcutaneously to the mice in the third group at a dose rate of 25 mg/kg. CA was administered orally either alone at a dose rate of 100 mg/kg or combined with a subcutaneous dose of DA at a dose rate of 50 mg/kg CA and 12.5 mg/kg DA to the mice in the fourth and fifth groups, respectively. The inhibitory impacts of each specific drug on *B. microti* growth were examined as described previously [18].

### Anemia monitoring in treated mice

The hematological parameters (HCT, hemoglobin [HGB], and RBC counts) were determined in the blood of all mice each 96 h utilizing Celltac a MEK-6450 electronic hematology analyzer (Nihon Kohden Enterprise, Tokyo, Japan).

### Statistical analysis

GraphPad Prism (version 5.0 for Windows; GraphPad Software, Inc., San Diego, CA, USA) was used to calculate the significant differences between examined groups using a one-way analysis of variance test. p<0.05 was considered statistically significant.

## Results

### CA methanolic extract represses the *in vitro* development of *Babesia* and *Theileria*

**Figure-1:**
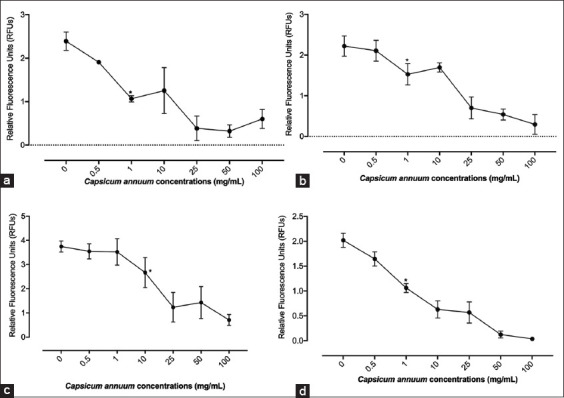
Inhibitory effect of *Capsicum annuum* on *Babesia bovis*, *Babesia divergens*, *Theileria equi*, and *Babesia caballi* on the 4^th^ day of treatment. (a) *B. bovis*. (b) *Babesia bigemina*. (c) *T. equi*. (d) *B. caballi*. Each value represents the mean±standard deviation of triplicate trials after subtracting the background fluorescence for non-parasitized red blood cells. Asterisks indicate a significant difference (analysis of variance; *p<0.05) between the *Capsicum annuum*-treated and the control cultures.

The calculated IC_50_s and IC_90_s revealed that the CA mainly affects the growth of *B. caballi*, and *B. bovis* followed by *T. equi* (Tables-1 and S1). *B. divergens* presented the most elevated IC_50_ among screened parasites (Tables-1 and S1). The *in vitro* development of *B. bovis*, *B. divergens*, and *B. caballi* was collectively repressed (p<0.05) by 1 mg/mL CA (Figure-1a-d). Moreover, 10 mg/mL of CA treatment collectively restrained (p<0.05) *T. equi* development (Figure-1c). The *in vitro* regrowth of *B. bovis*, *B. divergens*, and *T. equi* was repressed in the resulting viability test at a concentration of 25 mg/mL, 100 mg/mL, and 50 mg/mL CA, individually (Table-2). Notably, *B. caballi* treatment *in vitro* culture with 10 mg/mL CA forestalled parasite regrowth (Table-2). DA halted the regrowth of screened piroplasm parasites at 0.25 m/mL (Table-S2). The noticed non-significant contrast (p>0.05) between the positive control well containing the DMSO and untreated wells, showing that the diluent did not impact the CA methanolic extract viability. In addition, erythrocyte pre-treatment with a high centralization of CA methanolic extract 25 mg/mL does not influence either the parasite development example or erythrocyte morphology when contrasted with the non-treated erythrocytes by light magnifying instrument (data not shown).

**Table 1:** IC_50_ values of *Capsicum annuum* evaluated for bovine *Babesia* and equine *Babesia* and *Theileria* parasites

Organism	IC_50_ (mg/mL)^a^
*B. bovis*	4.87±1.23_(0.25)_
*B. divergens*	44.11±8.03_(0.18)_
*T. equi*	8.23±2.54_(0.31)_
*B. caballi*	1.26±0.50_(0.39)_

a IC_50_ values for *Capsicum annuum* was calculated based on the growth inhibitions determined using fluorescence-based assay in three separate experiments. Drug concentration was made in triplicate in each experiment, and the final obtained IC_50_ represents the mean and standard deviation of three separate experiments. The coefficient of variation data is shown as a subscript without a multiplication step.

**Table 2:** Viability test results of *Capsicum annuum* evaluated for bovine *Babesia* and equine *Babesia* and *Theileria* parasites

Drug	Drug concentrations (mg/mL)^a^						

	PC	0.5	1	10	25	50	100
*B. bovis*	254.11±73.50	252.07±68.42	249.82±63.66	265.41±44.56	91.23±23.08*	85.55±11.07*	21.08±8.11*
*B. divergens*	301.67±45.20	304.51±30.15	299.33±35.41	294.33±34.11	289.22±24.90	299.11±33.20	79.34±21.30*
*T. equi*	345.55±65.33	332.11±52.04	342.67±48.52	344.22±39.67	330.08±42.57	132.44±22.36*	45.31±17.88*
*B. caballi*	204.01±23.56	205±20.11	202.21±24.30	103.23±9.18*	91.33±15.43*	51.37±12.35*	12.87±4.09*

a Each value was calculated using fluorescence-based assay in three separate experiments. Each concentration of the drug was made in triplicate in each experiment, and the final obtained fluorescence value represents the mean and standard deviation of three separate experiments after subtraction of the background fluorescence for non-parasitized RBCs and multiplied by 100.

* *P<*0.05 statistically significant differences between the *Capsicum annuum* -treated and control groups.

### DA improved the *in vitro* adequacy of CA methanolic separate

Several combinations of CA with DA on piroplasm parasites indicated the low *in vitro* susceptibly to CA (*B. divergens* and *T. equi*) among screened parasites have been assessed. CA/DA mix showed higher inhibitory effects on *B. divergens*, and *T. equi* development than those noticed using DA monotherapy at M5 (0.25 IC_50_ CA:0.75 IC_50_ DA) and M6 (0.25 IC_50_ CA:0.50 IC_50_ DA), individually (Table-3). Such findings confirmed the potential anti-piroplasm impact of CA, especially when administered in lower dosages with the regularly used antibabesial drug DA.

**Table 3:** Growth inhibition effect of *Capsicum annuum*/diminazene aceturate combinations on *Babesia divergens* and *Theileria. equi*

Group	Fluorescence values (mean±SD)	

	*B. divergens*	*T. equi*
Control	243.05±7.10 _(0.03)_	325.15±12.34 _(0.04)_
DA IC_50_	120.11±6.43 _(0.05)_	152.11±4.01 _(0.03)_
M1 (—: —)	15.24±0.97**_(0.06)_	3.22±0.98**_(0.30)_
M2 (—: ½)	19.21±4.19**_(0.22)_	9.14±0.13 **_(0.01)_
M3 (½: —)	22.12±3.18**_(0.14)_	11.34±1.13**_(0.10)_
M4 (½: ½)	37.17±2.14**_(0.06)_	20.18±6.13**_(0.30)_
M5 (¼ : —)	80.16±3.21**_(0.04)_	29.33±5.12**_(0.17)_
M6 (¼: ½)	135.56±5.15*_(0.04)_	51.11±4.13**_(0.08)_
M7 (1/8 : —)	132.44±3.23*_(0.02)_	149.12±5.16*_(0.03)_
M8 (1/8 : ½)	140.11±4.12*_(0.03)_	156.39±7.50*_(0.05)_

* p<0.05 statistically significant differences between the combined-drug-treated and control groups only.

** P*<*0.05 statistically significant differences between the combined-drug-treated group and both the diminazene aceturate and control groups. M1–8 refer to the combinations of *Capsicum annuum*: DA, diminazene aceturate. The coefficient of variation data is shown as a subscript without a multiplication step.

### CA/DA clears *B. microti* infection in mice

**Figure-2:**
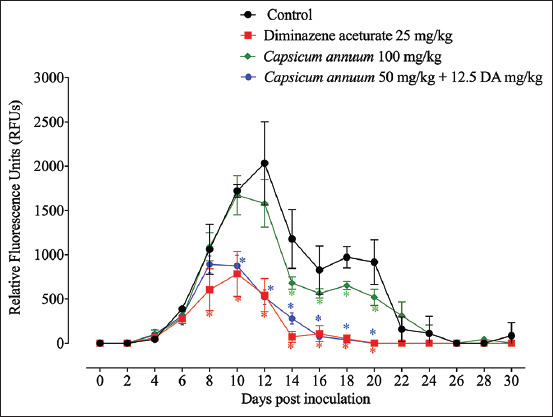
*In vivo* chemotherapeutic efficacy of *Capsicum annuum*, diminazene aceturate, and the combination of both drugs on the growth of *Babesia microti* estimated by the fluorescence assay. Each value represents the mean±standard deviation of five mice per experimental group. Asterisks indicate significant differences (analysis of variance; *p<0.05) between the treated and control groups.

**Figure-3:**
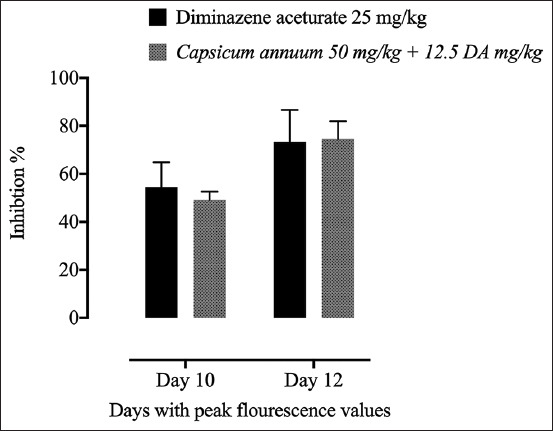
Percentages of inhibition in the growth of *B. microti* in mice caused by diminazene aceturate (DA), *Capsicum annuum/*DA on the days with peak parasitemia. The % of parasite inhibition in each treated group was calculated as a ratio to the positive control group.

The *in vivo* inhibitory effect of the CA was considered in contrast to *B. microti* in a mouse model. In correlation with the positive benchmark group, a considerable hindrance (p<0.05) in the produced fluorescence signals was observed in CA monotherapy-treated mice on days 14, 16, 18, and 20 p.i. (Figure-2). Notably, the inhibition within the fluorescence signals was comparable in mice treated with CA/DA combination and those from mice treated with ful l dose (25 mg/kg) DA at multiday p.i. (Figure-2). Oral administration of 50 mg/kg CA, when administered along with a subcutaneous dose of 12.5 mg/kg DA, caused 49.17% and 74.48% inhibition rates in *B. microti* growth on 10 and 12 days pi, respectively. In contrast, 25 mg/kg DA monotherapy showed 54.46% and 73.42% inhibition of parasite growth on days 10 and 12 pi (Figure-3).

### CA/DA treats hemolytic sickliness brought about by *B. microti* infection in mice

**Figure-4:**
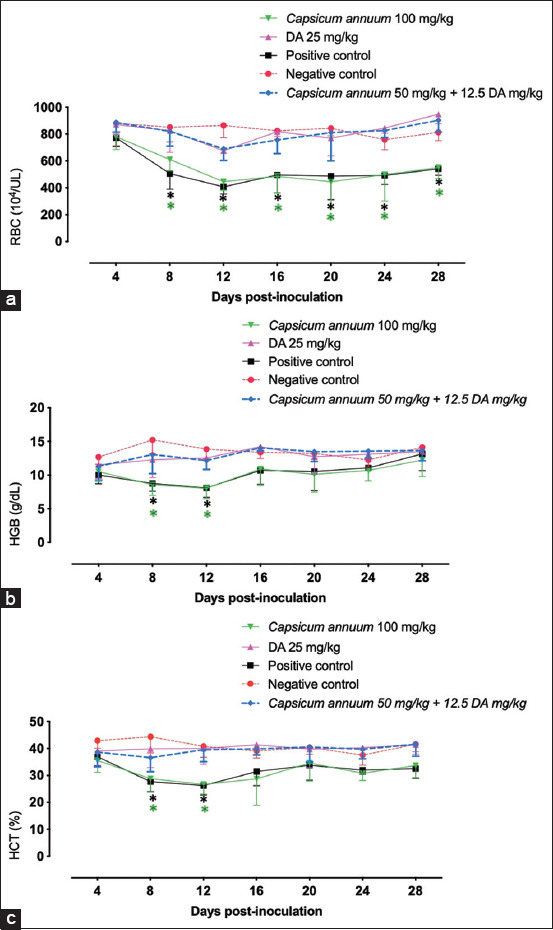
Hematological variables in *Babesia microti*-infected mice treated with *Capsicum annuum*. (a) Red blood cells. (b) Hemoglobin. (c) Hematocrit. Each value represents the mean ± standard deviation of five mice per experimental group. Asterisks indicate a significant difference (analysis of variance; *p<0.05) between the treated or infected mice and the uninfected mice.

Interestingly, treatment of mice with CA and a low portion of DA standardized the evaluated hematological factors practically like those treated with 25 mg/kg DA (Figure-4). Contrastly, a significant decrease (p<0.05) in RBCs included in mice treated with CA monotherapy in examination with negative control mice (got neither disease nor treatment) on days 4-24 p.i. like those detected in positive control mice (Figure-4a). Comparatively, in examination with negative control mice, a significant decrease (p<0.05) in HGB level and HCT % on days 8 and 10 in mice treated with CA monotherapy (Figure-4b and c). Such discoveries showed the promising antibabesial adequacy of CA/DA mix treatment.

## Discussion

Extraction is the most common method for extracting bioactive chemicals from biomass materials. The extraction procedure aims to obtain the maximum biological activity from the extracts while maximizing the number of target components. The extraction solvent and the extraction technique have an impact on the extraction yield and biological activity of the final extract[19,20]. Many solvents have been used to extract bioactive chemicals from plant material, including methanol, ethanol, acetone, and water. Because of the diversity of bioactive chemicals found in plant materials and their varying solubility qualities in several solvents, the best solvent for extraction is determined by the plant materials and the compounds to be extracted. Truong *et al*. [20] reported that methanol is the most effective solvent for obtaining an extraction from *Severinia buxifolia* over other solvents, including distilled water, ethanol, chloroform, dichloromethane, and acetone. Using methanol resulted in the highest extraction yield (33.2%) and the highest content of phenolic, flavonoid, alkaloid, and terpenoids from *Severinia buxifolia*. In addition, the extract obtained from methanol showed high antioxidant capacity and *in vitro* anti-inflammatory activity [20]. Subsequently, those authors recommended methanol as the optimal solvent to obtain high content of phytochemical constituents and high antioxidants constituents for usage in pharmacognosy. Therefore, in this study, we evaluated the inhibitory effect of CA’s methanol extract on *B. bovis*, *B. divergens*, *T. equi*, and *B. caballi* *in vitro* and *B. microti* development *in vivo*.

*B. caballi* and *B. bovis* were the touchiest to CA, trailed by *T. equi* and *B. divergens*. The IC_50_ upsides of CA for *Babesia* and *Theileria* parasites were higher than those of recently screened restorative plants, allicin [21], fusidic acid [22], and thymoquinone [17]. A significantly high concentration of CA did not affect bovine or horse RBCs in the current examination. This information support CA’s non-harmful properties. The issue shows CA’s safety *in vitro* tries and suggests that this promising agent of piroplasm up-and-comer be used *in vivo* examinations.

Although the *in vitro* development of *Babesia* and *Theileria* parasites was considerably restrained within sight of a high convergence of CA, its inhibitory effects were enhanced when combined with DA. These outcomes are like the *in vitro* inhibitory effects of myrrh oil/DA [13], allicin/DA [21], and TQ/DA combinations [17]. These findings propel us to research the inhibitory effect of CA/DA in mice infected with *B. microti*. Interestingly, the inhibition in *B. microti* development induced by CA/DA is higher than 70% inhibition rates for clindamycin combined with quinine [23], 56.35% and 53.25% inhibition rates for 85 mg/kg PYR/10 mg/kg DA [18], 67% inhibition rates for 50 mg/kg enoxacin and 10 mg/kg DA [4], and 62.5% inhibition rates for 50 mg/kg oral dose of TQ and 10 mg/kg subcutaneous dose of DA [17].

CA is known as a potent antioxidant agent [8]. Reactive oxygen and nitrogen species (ROS and RNS, respectively) have recently been shown to play a role in the pathogenesis of parasite diseases [24]. Furthermore, parasitic infestations are followed by various oxidant-generating enzymes, which drive the development of various inflammatory cells, which then eliminate intracellular and extracellular parasites [25]. By nitration, oxidation, and chlorination processes, these ROS and RNS are mainly produced to combat invading microorganisms. However, excessive levels of similar reactions might harm host cells and tissue [25]. Infection with *Babesia* increases the generation of free radicals and oxidative stress markers such as malondialdehyde, protein carbonyl content, and plasma concentrations of nitric oxide (NOx) metabolites and lowers total antioxidant activity and glutathione levels [26]. Therefore, the CA extract’s antibabesial adequacy may be attributed to the potent antioxidant effect of CA. Although this study evaluated the inhibitory effect of methanolic extract of CA against the growth of piroplasm parasites, the study neglected the use of hydroalcoholic extract of CA, which will help in obtaining the polar and non-polar compounds of CA. Therefore, future studies are required to evaluate the inhibitory effect of hydroalcoholic extract of CA against the growth of piroplasm parasites either *in vitro* or *in vivo*.

## Conclusion

*B. caballi* and *B. bovis* were the most susceptible parasite to the *in vitro* inhibitory activity of CA. The combination of CA and DA was found to have a potent inhibitory effect on the *in vitro* growth of *B. divergens* and *B. caballi*. Mice treated with a combination treatment containing lower doses of CA and DA showed an extensive decrease in fluorescence levels. Besides, the CA/DA combination therapy could treat the hemolytic anemia associated with *B. microti* in mice. Such findings show that the CA might be compelling in treating piroplasmosis, especially when combined with a low dose of DA (12.5 mg/kg).

## Authors’ Contributions

MAR, SAEE, and II: Conceptualization. MAR: Data curation. MAR and SAEE: Formal analysis. MAR, II, and MA: Funding acquisition. MAR and II: Investigation. MAR, SAEE, and MA: Methodology. II: Project administration. MAR and II: Resources. MAR: Software. II: Supervision. MAR and II: Validation. MAR and II: Visualization. MAR, SAEE, and MA: Writing of original draft. All authors read and approved the final manuscript.

## Appendix

**Supplementary data**

**Table S1:** IC_90_ values of *Capsicum annuum* evaluated for bovine *Babesia* and equine *Babesia* and *Theileria* parasites.

Organism	IC_90_ (mg/mL)^a^
*B. bovis*	8.77
*B. divergens*	79.40
*T. equi*	14.81
*B. caballi*	2.27

a IC_90_ values for *Capsicum annuum* were calculated based on the IC_50_ values obtained from *in vitro* growth inhibition assay. *B. bovis=Babesia bovis*, *B. divergens=Babesia divergens*, *B. caballi=Babesia caballi*

**Table S2:** Viability test results of diminazene aceturate drug evaluated for *Babesia* and *Theileria* parasite.

Drug	Drug concentrations (μg/mL)^a^				

	10	5	1	0.5	0.25
*B. bovis*	−	−	−	−	−
*B. divergens*	−	−	−	−	−
*T. equi*	−	−	−	−	−
*B. caballi*	−	−	−	−	−

a Each value was calculated using *Babesia* fluorescence assay ( *B*FA) in three separate experiments. Each concentration of the drug was made in triplicate in each experiment. +=viable; −=dead. *B. bovis=Babesia bovis*, *B. divergens=Babesia divergens*, *B. caballi=Babesia caballi*, *T. equi=Theileria equi*
